# Adverse Effects of Sinopharm COVID-19 Vaccine among Vaccinated Medical Students and Health Care Workers

**DOI:** 10.3390/vaccines11010105

**Published:** 2023-01-01

**Authors:** Anusha Sultan Meo, Adeeba Masood, Usama Shabbir, Hubba Ali, Zeeshan Nadeem, Sultan Ayoub Meo, Abdullah Nasser Alshahrani, Saad AlAnazi, Abeer A Al-Masri, Thamir Al-Khlaiwi

**Affiliations:** 1 College of Medicine, King Saud University, Riyadh 11461, Saudi Arabia; 2 Army Medical College, National University of Medical Sciences, Rawalpindi 46000, Pakistan; 3 Department of Physiology, College of Medicine, King Saud University, Riyadh 1146A1, Saudi Arabia

**Keywords:** Sinopharm, vaccine, adverse effects, COVID-19 pandemic

## Abstract

The severe acute respiratory syndrome coronavirus 2 (SARS-CoV-2) disease caused a highly problematic situation worldwide. Various vaccines were launched to combat the COVID-19 pandemic. This study aims to investigate the adverse effects of first and second doses of the Sinopharm vaccine among vaccinated medical and dental students and healthcare workers. A well-established questionnaire was distributed online, and 414 medical and dental students and healthcare workers (HCW) comprising 355 females (85.7%) and 59 males (14.3%) participated; all were vaccinated with two doses of Sinopharm. The most common side effect was pain at the injection site after dose one in 253 respondents (61.3%) and after dose two in 161 respondents (38.9%). Other symptoms included general lethargy in 168 (40.6%), myalgia/body pain in 99 (23.9%), low-grade fever in 93 (22.4%), and headache in 87 (21%) respondents. Common side effects reported after the second dose of the vaccine following pain at the injection site included general lethargy in 21.3% (88), headache in 10.4% (43), myalgia/body pain in 9.9% (41), and low-grade fever in 6.1% (25) of the respondents. In conclusion, common adverse effects of the Sinopharm vaccine were pain at the injection site, general lethargy, myalgia, body pain, low-grade fever, and headache. These adverse effects were mild in intensity for both doses but slightly more frequent and severe for the first dose than the second dose.

## 1. Introduction

Severe acute respiratory syndrome coronavirus-2 (SARS-CoV-2), also known as the COVID-19 pandemic, has swiftly spread in a matter of months across the world. The outbreak of the disease has posed a major threat to international healthcare systems and economies [1]. On 22 December 2022, the disease infected 651,918,402 people and caused 6,656,601 deaths with a case fatality rate of 1.02% [2]. Worldwide, most countries have witnessed about four waves of the deadly pandemic, and the disease caused various clinical manifestations in the general population [3,4,5]. SARS-CoV-2 is a highly contagious disease and spreads with mutable prevalence and mortality outbreak trends [6,7]. The vaccination is highly beneficial to reduce the risk of the infection, severity, and mortality of the disease and is considered a long-term solution to eradicating SARS-CoV-2 [8,9,10].

In a two-year period from December 2020 to December 2022, from the date of launching the COVID-19 vaccine, 68.7% of the global population has received at least one dose of a COVID-19 vaccine worldwide; 13.09 billion doses have been administered globally, and 2.98 million are now administered per day. However, in low-income, developing countries, only 25.1% of people have received at least one dose of a COVID-19 vaccine [11]. The emergence of SARS-CoV-2 new variants threatens global prevention efforts [12] and the impact of mass vaccination campaigns against the COVID-19 pandemic.

Despite the evidence for the safety and effectiveness of vaccines, there is still hesitancy about their uptake in diverse cultural contexts. The ignoring and refusing vaccination against COVID-19 caused an extensive burden on healthcare systems and economies and pose a risk to global health [13]. This behavior is seen in various ages and professions, including healthcare workers [14,15].

In the countries with high vaccine coverage, there have been massive reductions in SARS-CoV-2 disease, hospitalization, and death, but globally, vaccine access is highly inequitable with coverage ranging from 1% to over 70% depending largely on the country’s wealth and policies. Consequently, SARS-CoV-2 variants continue to emerge, causing surges in the disease [16]. Global climate change, natural resources depletion, and wars are some of the great global challenges of this time in which SARS-CoV-2 is spreading, and that can influence vaccine strategies [17]. Achieving global COVID-19 vaccination targets needs urgent action across regional, national, and global levels to improve the supply of COVID-19 vaccines to low- and middle-income countries [16].

Many COVID-19 vaccine candidates have been developed by major global biopharmaceutical companies. Sinopharm, also known as the BBIBP-CorV vaccine, is one of two inactivated virus COVID-19 vaccines. Sinopharm obtained an emergency use authorization (EUA) in July 2021. Initially, Sinopharm was approved for use in Asia, Africa, and the Middle East [18,19]. Two doses of Sinopharm have an efficacy of 79% [20]. Similar to every other drug or vaccine that promises cure and prevention, it can have adverse effects. The fact is that limited research literature exists on the Sinopharm vaccine and its side effects, despite it being a commonly used vaccine in some countries. This study aims to investigate the adverse effects of the Sinopharm vaccine among medical and dental students and healthcare workers.

## 2. Subjects and Methods

### 2.1. Study Design and Settings

This cross-sectional study was carried out under the supervision of the Department of Physiology, College of Medicine, King Saud University, Riyadh and National University of Medical Sciences, Rawalpindi, Pakistan. A well-structured online questionnaire was established to achieve the study objectives.

### 2.2. Study Participants

The study participants were medical and dental students and healthcare workers, including physicians, dentists, and paramedical staff, who had received both doses of the Sinopharm vaccine. The second dose was received 28 days after the first dose. The questionnaire was circulated after a substantial gap of 2 months after both doses. Initially, the two investigators visited medical and dental schools, postgraduate medical centers, student councils, and health administration offices in twin cities of Rawalpindi and Islamabad, Pakistan to collect the contact details of medical and dental students and healthcare workers. After obtaining the contact details, a message was sent for their consent and voluntary participation in this study.

### 2.3. Inclusion and Exclusion Criteria

We included participants who were medical or dental students or healthcare workers, who provided informed consent, and who had received both doses of the Sinopharm vaccine. The exclusion criteria included those who were not medical or dental students, who were not healthcare professionals, who had not received a double dose, who had been vaccinated by any other vaccine besides Sinopharm, or who had not been vaccinated for COVID-19 in the first place.

### 2.4. Sample Size Calculation

The minimum sample size for this study was calculated following the study protocol [21] and using the “Raosoft” power calculator [22]. In the metropolitan cities of Rawalpindi and Islamabad, Pakistan, regional authorities aim to vaccinate 70% of the population, approximately 35,000 individuals, and a sample size of 380 participants was sufficient to achieve 95% confidence with a 5% margin of error. However, in the present study, 414 medical and dental students and healthcare workers participated.

### 2.5. Study Questionnaire Development

An English-language, self-administered, online web-based questionnaire was developed after extensive review of the literature on adverse events of various COVID-19 vaccines [23]. The final versions of the questionnaire were reviewed by three different faculty members to check the face and content validity. The questionnaire was circulated via a link survey to different platforms on social media and other mailing platforms, such as email and WhatsApp [23,24]. It was initially distributed to a pilot sample consisting of 10 faculty members to countercheck the validity of the questionnaire and for any technical concerns. We attempted to make the survey as brief and to the point as possible. We explained that the information provided would be only utilized for research purposes because the participants were not identified and had the right to withdraw at any stage. The survey consisted of an initial page for informed consent, and all contributors were allowed to freely choose whether or not to participate. No reward or incentive whatsoever was given to the participants, and the information was kept entirely confidential. The validated, self-administered, electronic questionnaire was then distributed via email, WhatsApp, and social media groups.

The questionnaire contained 31 questions and was divided into 8 sections. The first section regarded the consent of the medical and dental students and healthcare workers to participate and their willingness to share their responses for this study with no incentives provided whatsoever. The second section consisted of sociodemographic information, such as gender and age range. The third section asked whether the participant had any systemic diseases/comorbidities or allergies. The fourth section regarded COVID-19 vaccination details, including the dates of both dosages and the site of vaccination. Section five was about COVID-19 history, including when the disease was contracted, for how many days the infection lasted, whether it was contracted before or after COVID-19 vaccination, and, if so, how many days after vaccination. The sixth section pertained to twenty-five adverse effects postvaccination, which were listed identically for both doses 1 and 2. Participants were asked to select all the relevant side effects they experienced after vaccination, respectively, for both doses. The seventh and eighth sections were about individual details of each of the doses and their side effects in terms of onset, the duration for which they persisted, their severity on a scale of 1–5, and whether they required medication and/or hospital admission.

Most questions were closed-ended except when questions were asked regarding the history of COVID-19, details of testing positive in terms of the number of days, or any complications that required hospitalization. In the case of any additional adverse effects of vaccinations experienced by any participant that were not mentioned in the list, individuals were allowed to mention it separately in response to an open-ended question. The questionnaire had an introductory paragraph explaining the nature and objectives of the study and the voluntary and anonymous nature of participation.

### 2.6. Ethical Statement

The Institutional Review Board, College of Medicine, King Saud University, Research Centre approved the study (Ref. E-22-7234).

### 2.7. Statistical Analysis

Statistical analysis was done by using the software Statistical Package for Social Sciences (IBM-SPSS) version 25 (New York, NY, USA). Descriptive statistics were calculated in form of frequencies and percentages to describe the study samples, vaccines, and adverse events. After this, significant associations of the variables with adverse events of the vaccines and cross-tabulation with the Chi-square method were used. A general linear model (multivariate analysis of variance, MANOVA) was conducted to inspect significant differences in adverse events.

## 3. Results

In this cross-sectional study, four-hundred and fourteen (414) people participated, all of whom were vaccinated with both doses of Sinopharm, where the majority of respondents were males at 85.7% (355), and 14.3% (59) were females. A total of 96.4% (399) of the vaccine recipients belonged to the age group between 18 and 25 years, 2.2% (9) fell in the range of 26–35 years, 0.2% (1) in the 46–55 years range, and 1.2% (5) were between 56 and 65 years.

Out of the 414 vaccine recipients, 16.7% (69) of people had a history of allergies. The most reported comorbidity was asthma at 5.07% (21) followed by hypertension at 1.69% (7), ischemic heart disease at 0.72% (3), and diabetes mellitus at 0.48% (2). No one reported a history of tuberculosis.

When the COVID-19 history was taken, 79.5% (329) of the participants had no previous COVID-19 history while 20.5% (85) people had contracted the virus in the past. Amongst those who had been infected previously, 82.4% of these eighty-five individuals (70) contracted it before getting vaccinated with Sinopharm while 17.6% (15) contracted it after getting vaccinated. The respondents’ sociodemographic data are summarized along with their history of COVID-19 infections in Table 1.

Out of the twenty-five potential adverse events that were listed for each dose, the results revealed that the most common adverse event after both dose one and dose two was “pain at injection site”, reported by 61.3% (253) and 38.9% (161), respectively. For dose one, in order of precedence of frequency, other symptoms included general lethargy in 40.6% (168), myalgia/body pain in 23.9% (99), low-grade fever in 22.4% (93), and headache in 21% (87) of the respondents. No side effects were encountered at all in 19.3% (80) of participants. The most common side effects reported postdose two of the vaccine following pain at the injection site were general lethargy in 21.3% (88), headache in 10.4% (43), myalgia/body pain in 9.9% (41), and low-grade fever in 6.1% (25) of respondents while no side effects were seen in 54.3% (225). In total, no overall side effects from any dose were reported in 13.8% of the respondents (57) (Table 2).

**Figure 1:**
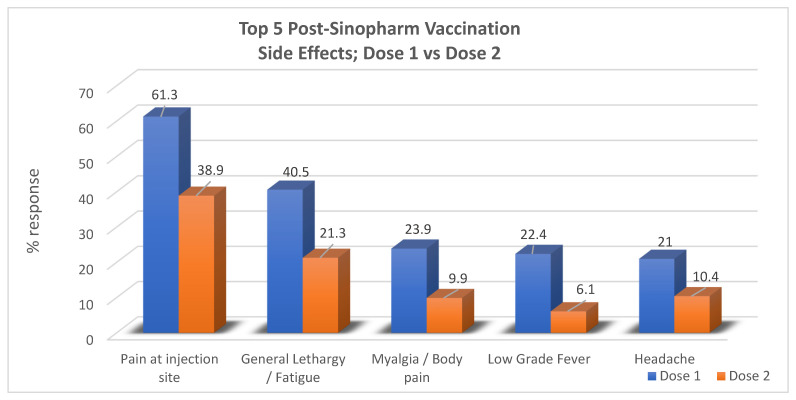
Comparison between the outcome regarding adverse effects for dose one and dose two of the Sinopharm vaccine.

When the results of dose one and dose two were compared, it was noted that most of the participants experienced a greater number of symptoms in the form of a larger total number of postvaccine symptoms (out of 25 symptoms) for dose one than they did for dose two. Moreover, each side effect was reported by a greater number of participants for dose one compared to dose two, as pictorially represented in the bar graph below (Figure 1).

The onset of symptoms postvaccination dose one and dose two was observed by most participants within 1–4 h by 30.7% (127) of respondents after dose one and by 18.6% (77) of participants after dose two. After dose one, postvaccination symptoms persisted most commonly for 1–2 days in 33.8% (140) of individuals, which was similar to dose two, in which symptoms persisted for 1–2 days as well as shown by responses from 22.7% (94) of the participants. The results also revealed that the severity of the adverse effects was more for dose one, where 24.60% (102) individuals selected the option for severity level 2, as compared to dose two, where the most frequent option selected was severity level 1 by 25.10% (104) of the participants. After the first dose of the Sinopharm vaccine, 24.2% (100) people required medication to relieve the symptoms as compared to dose two, where a lower frequency of 14.5% (60) of individuals required medication for relief. A greater number of people also reported feeling unable to work after dose one, 24.6% (110), as compared to 13.0% (54) of respondents who complained similarly for dose two. The results for the presentations of the most reported symptoms following Sinopharm vaccination dose one and dose two are summarized in Table 3.

The association between variables was analyzed. There was no significant correlation between the total score of side effects out of 50 with the age range (*p* = 0.252). No association was seen between gender and the side effects score (*p* = 0.632); no correlation was seen between side effects and comorbidities (*p* = 0.496). However, individuals with a previous COVID-19 history did show an association with the number of total symptoms experienced postvaccination (*p* = 0.011). The severity of the side effects for both doses (1–5) was not associated with gender (*p* = 0.460) (Table 4).

## 4. Discussion

COVID-19 vaccinations have altered the course of the pandemic, saved tens of millions of lives globally, and have also decreased disease-related morbidity and disabilities worldwide [25]. Vaccination protects individuals and communities by reducing the spread of diseases within a population, and vaccines are the world’s safest tools to protect people from life-threatening diseases. During the global outbreak of the COVID-19 pandemic, vaccination played a vital role in minimizing COVID-19 cases and deaths [26]. However, worldwide, people have some safety concerns about vaccinations. The fact is that to date, extremely limited research literature exists on the Sinopharm vaccine and its side effects despite it being the most widely administered COVID-19 vaccine in some countries. Once such studies are published from various countries, it will be possible to provide more accurate fact-based conclusions, and it will be much easier to establish future guidelines [27]. The present study showed that the most common adverse effects after both dose one and dose two were pain at the injection site, general lethargy, myalgia, body pain, low-grade fever, and headache. The participants experienced symptoms with greater severity and greater frequency of side effects following dose one than dose two. The onset of symptoms postvaccination with dose one and dose two was observed by most participants within 1–4 h and persisted most commonly for 1–2 days.

Sinopharm obtained authorization in July 2021 as one of many vaccines to control the ongoing pandemic by providing immunity against the infection [28]. Morbidity and mortality from COVID-19 in certain susceptible members of the population at a substantial risk for morbidity or mortality are significant risks, and the treatment options for some of these people are limited. Widespread vaccination programs to prevent infection, while associated with some minimal postvaccination symptoms, serve as a valuable tool in efforts to control the pandemic [29].

The present study results are consistent with a study conducted in the UAE which also aimed at evaluating the most common side effects of Sinopharm [30]. It showed that for the first dose, the most common side effects among the participants were pain at the site of vaccination (42.2%), fatigue (12.2%), and headache (9.6%). There were no side effects for 24.4% of the participants. For the second dose, the most common side effects were pain at the vaccination site (32.6%), fatigue (16.3%), and lethargy (13.7%). No side effects were reported by 14% of the participants [28]. A difference noticed in this study compared to our present study is that our results revealed that headache was not among the top three side effects after the first dose, unlike in their study.

In the USA, research has been conducted to investigate the side effects following a Pfizer COVID-19 vaccination. The main generalized symptoms that were reported were generalized weakness or fatigue (58.9%), headache (44.8%), chills (36%), and fever (22%) [31]. Approximately 88% of healthcare workers reported a sore arm or pain at the injection site as their primary localized side effect followed by localized swelling at the injection site (5.5%), itching (5.4%), lymphadenopathy (axillary or regional) (3.4%), and skin rashes 20 (2.49%). For the participants who received a Pfizer vaccination, swelling at the site of injection was the second most common symptom that occurred, which was not seen in our study as one of the common side effects. No such adverse effects were noted in our study with Sinopharm. Similarly, a study conducted on Pfizer, AstraZeneca, and Sinopharm in Iraq [32] showed the following results regarding the Sinopharm vaccine’s side effects: injection site reaction (54.5%), fatigue (40.9%), fever (37.8%), myalgia (36.3%), and headache (33.3%).

Similarly, Thonginnetra et al., 2022 [33] conducted a study in Thailand and assessed the vaccine safety among participants who were vaccinated. The participants experienced pain at the injection site and tenderness (37.93%), fatigue (37.89%), myalgia (33.56%), and headache (26.76%) as the most common clinical adverse effects. The authors further reported that two doses of the BBIBP-CorV caused mild-to-moderate side effects in adolescents in Thailand. Similarly, Zahid, 2021 [34] conducted a study and investigated the side effects resulting from the first and second doses of different vaccines used in Bahrain. The author observed that recipients of the Sinopharm vaccine reported the mildest side effects among all four vaccines. 

Dar-Odeh et al., 2022 [35] conducted a study in Jordan and Saudi Arabia and reported that the most frequently administered vaccines were “Pfizer-BioNTech, Sinopharm, and AstraZeneca” vaccines. The participants reported the adverse effects, which were mainly “fatigue, menstrual disturbances, myalgia, arthralgia, dizziness, and headache.” There was no statistically significant association between age, gender, or medical history with adverse events. The collective symptoms of “fatigue, myalgia, arthralgia, dizziness, and headache” were significantly associated with the Sinopharm vaccine. All these studies conducted in different countries highlighted various adverse events of COVID-19 vaccination. Similarly, the present study findings showed more or less the same adverse effects of pain at the injection site, general lethargy, myalgia, low-grade fever, and headaches as the most common side effects of the Sinopharm vaccine.

## 5. Strength and Limitations

The strengths of our study include the fact that to date, extremely limited research literature exists on the Sinopharm vaccine and its side effects despite it being the most widely administered vaccine in some countries. A limited literature search was conducted on Sinopharm worldwide; therefore, we found it essential to conduct a study to have thorough knowledge of the presenting adverse effects, thereby ensuring that this vaccine is safe for the population and has no severe adverse effects. The participants involved in this survey were medical and dental students and healthcare workers. Being from the medical field, these individuals are well-educated about the vaccine and its side effects and given their nature of work in hospitals, are under constant exposure to the virus, thereby data from such a population gives a great estimate of its efficacy ensuring if the vaccine renders individuals safe from the virus. The limitations of the study include the small sample size, given the circulation of the form’s online-based link self-administrated questionnaire. This is a cross-sectional study based on self-reported adverse effects, which could be influenced by participants’ prior prejudice and misinformation about vaccines. The survey was filled out by medical students, and the age range data was limited to a young age group of healthier, mainly males, who also do not suffer from significant comorbidities. The adverse effects that occurred may be at some level ignored.

## 6. Conclusions

The most common adverse effects of the Sinopharm vaccine following both doses are pain at the injection site, general lethargy, myalgia, low-grade fever, and headache. The postvaccination adverse effects of the Sinopharm COVID-19 vaccine following the first and second doses were mild, predictable, and non-life-threatening. These adverse effects were mild in intensity for both doses but slightly more frequent and severe for the first dose than the second dose. The Sinopharm vaccine has a mild degree of adverse effects and can be considered a safe vaccine for future use.

## 7. Study Implications

The present study provides valuable information on the adverse effects of the Sinopharm vaccine among medical and dental students and healthcare workers. There is a dire need for such studies from various parts of the world for tracking the safety of the Sinopharm vaccine, providing feedback to the public and policymakers, improving public trust in the vaccine’s safety, and eradicating the stigma in society regarding the vaccine’s safety for combatting the COVID-19 pandemic. The present study results may enhance public confidence both at regional and international levels by dispelling misconceptions and conspiracy theories concerning postvaccination adverse effects of COVID-19 vaccines.

## Figures and Tables

**Table 1:** Sociodemographic data along with the statistics of the COVID-19 history of participants.

Parameter	Frequency (#)	Frequency (%)
Gender		
Male	355	85.7
Females	59	14.3
Age (years)		
18–25	399	96.4
26–35	9	2.2
36–45	0	0
46–55	1	0.2
56–65	5	1.2
Comorbidities		
Allergies	69	16.70
Asthma	21	5.07
Hypertension	7	1.69
Ischemic Heart Disease	3	0.72
Diabetes Mellitus	2	0.48
Tuberculosis	0	0
History of COVID-19		
Positive	85	20.5
Negative	329	79.5
If positive, was COVID-19 contracted before or after vaccination		
Before vaccination	70	82.4
After vaccination	15	17.6

**Table 2:** The most common adverse effects postdose one and dose two of Sinopharm vaccination.

DOSE 1		DOSE 2	
Top 5 Symptoms	Percentage/Frequency	Top 5 Symptoms	Percentage/Frequency
1. Pain at the injection site	61.3% (253)	1. Pain at the injection site	38.9% (161)
2. General lethargy	40.6% (168)	2. General lethargy	21.3% (88)
3. Myalgia/body aches	23.9% (99)	3. Headache	10.4% (43)
4. Low-grade fever	22.4% (93)	4. Myalgia/body aches	9.9% (41)
5. Headache	21% (87)	5. Low-grade fever	6.1% (25)

**Table 3:** The most common presentation of how symptoms manifested after Sinopharm vaccination dose one vs. dose two.

Manifestation of Symptoms	Most Common for 1st Dose		Most Common for 2nd Dose	
Onset of symptoms	1–4 h	30.70%	1–4 h	18.60%
Duration of symptoms	1–2 days	33.80%	1–2 days	22.70%
The severity of symptoms (1–5)	Severity–2	24.60%	Severity–1	25.10%
Required medications (No/Yes)	No 75.8%	Yes 24.2%	No 85.5%	Yes 14.5%
Inconvenience to work (No/Yes)	No 75.4%	Yes 24.6%	No 87.0%	Yes 13.0%

**Table 4:** Summary of the association/significance of the correlation between different variables.

Parameter 1	Parameter 2	*p* Value	Significance
Age Range	Side effects score/50	0.252	Null hypothesis accepted (*p* => 0.05)
Gender	Side effects score/50	0.632	Null hypothesis accepted (*p* => 0.05)
Comorbidities	Side effects score/50	0.496	Null hypothesis accepted (*p* => 0.05)
COVID-19 history	Side effects score/50	0.011	Null hypothesis rejected (*p* =< 0.05)
Gender	The severity of vaccine side effects (both doses)	0.460	Null hypothesis accepted (*p* => 0.05)

## Data Availability

May be provided on reasonable request to the corresponding author.
